# Intrinsic and extrinsic factors drive ontogeny of early-life at-sea behaviour in a marine top predator

**DOI:** 10.1038/s41598-017-15859-8

**Published:** 2017-11-14

**Authors:** Matt I. D. Carter, Deborah J. F. Russell, Clare B. Embling, Clint J. Blight, David Thompson, Philip J. Hosegood, Kimberley A. Bennett

**Affiliations:** 1 School of Biological & Marine Sciences, Plymouth University, Plymouth, PL4 8AA UK; 2 Sea Mammal Research Unit, Scottish Oceans Institute, University of St Andrews, St Andrews, KY16 8LB UK; 3 Centre for Research into Ecological & Environmental Modelling, University of St Andrews, St Andrews, KY16 9LZ UK; 4 School of Science, Engineering & Technology, Abertay University, Dundee, DD1 1HG UK

## Abstract

Young animals must learn to forage effectively to survive the transition from parental provisioning to independent feeding. Rapid development of successful foraging strategies is particularly important for capital breeders that do not receive parental guidance after weaning. The intrinsic and extrinsic drivers of variation in ontogeny of foraging are poorly understood for many species. Grey seals (*Halichoerus grypus*) are typical capital breeders; pups are abandoned on the natal site after a brief suckling phase, and must develop foraging skills without external input. We collected location and dive data from recently-weaned grey seal pups from two regions of the United Kingdom (the North Sea and the Celtic and Irish Seas) using animal-borne telemetry devices during their first months of independence at sea. Dive duration, depth, bottom time, and benthic diving increased over the first 40 days. The shape and magnitude of changes differed between regions. Females consistently had longer bottom times, and in the Celtic and Irish Seas they used shallower water than males. Regional sex differences suggest that extrinsic factors, such as water depth, contribute to behavioural sexual segregation. We recommend that conservation strategies consider movements of young naïve animals in addition to those of adults to account for developmental behavioural changes.

## Introduction

Transition from dependence on parental provisioning to independent feeding is a critical time in the life of all animal species that receive parental care. For slow-maturing species, the first months of independent life are crucial in determining survival to recruitment, and therefore sustaining stable populations^1–3^. Survival depends on developing the ability to successfully find, compete for, capture and handle food resources whilst avoiding predation^4,5^. Juvenile behaviour, and its relationship with the development of successful feeding strategies, is receiving increasing research interest given the influence of early-life survival on population dynamics^1,3,6–9^. Unpicking the intrinsic and extrinsic factors that affect the development of foraging skills is key to understanding population trajectories and identifying critical habitat for species during their most vulnerable life stages.

For air-breathing marine diving predators, such as marine mammals, sea turtles, and seabirds, the challenge of developing effective foraging strategies is particularly acute. Individuals must locate and exploit patchily-distributed prey resources in a dynamic environment, within the physiological constraints of breath-hold diving^10^. Studying ontogeny in wild marine predators is problematic, not least because a considerable proportion of their lives is spent at sea, often underwater, where direct observations of behaviour are difficult or impossible^7^. Acoustic, satellite and Global System for Mobile communication (GSM) telemetry devices have allowed ecologists to track diving predators at sea, building an increasingly clear picture of their movements and dive behaviour^11,12^. Logistical and practical constraints, such as high mortality rates and low re-encounter probability, mean that behavioural datasets for young animals are sparse^13^. Pinnipeds and seabirds are dependent on terrestrial habitat for reproduction, and young animals are large enough to carry biologging devices, therefore providing tractable opportunities to record location and behavioural data spanning the initial months of independence^13^.

Many pinniped species, including otariids and walruses (odobenids), are income breeders^14^: they have protracted dependency periods, during which the young learn diving and foraging skills before weaning^15,16^. The nursing period may last many months, or even years^14^. Other pinnipeds (phocids), exhibit a range of breeding strategies. Some small phocids, such as harbour seals (*Phoca vitulina*), are also income breeders, but, in contrast to otariids, pups can dive within hours of birth. Despite short dependency periods (<1 month^17^), harbour seal pups can develop diving skills during suckling and may accompany their mothers on foraging excursions^18^. Synchronous diving of mothers and pups during lactation also occurs in some ice-breeding phocids^19^. Larger phocid species, such as elephant (*Mirounga spp*.), hooded (*Cystophora cristata*) and grey (*Halichoerus grypus*) seals, are usually capital breeders, and pups are abruptly abandoned at the natal site after a brief nursing period^14^. Grey seals, for example, suckle for 15–21 days^20^. Pups then undergo a post-weaning fast, usually on land, of between nine and 40 days, during which time they lose up to 25% of their body mass^21,22^. After departure from the natal colony, they must learn to dive and find food without maternal provisioning, or the benefit of observing the foraging behaviour of their mother^23^. Furthermore, they must do this before their remaining blubber and protein reserves are depleted to critical levels and terminal starvation begins^24^.

Swimming in cold water and diving to depth is energetically costly, and seal pups have a higher surface area to volume ratio, higher mass-specific metabolic rate and lower mass-specific oxygen storage capacity than adults^25,26^. In contrast to adults, young seals repeatedly dive up to their physiological limits and foraging efficiency is therefore lower because they must spend longer at the surface to recover^15,27^. Maximum diving capability increases in grey seal pups during the first months at sea^24^, but little is known about the development of their routine behaviours. First year mortality is high and variable between years for grey seals^28–30^, which has a profound effect on population dynamics^31^. Moreover, first year survival probability appears to be three times greater for females than males, regardless of body condition at weaning^29^. Differences in survival between male and female pups could be linked to development of sex-specific diving behaviour, leading to the sex difference in foraging strategies underpinned by sexual size dimorphism in adults^32^. In general, adult grey seals make repeated, short duration (3–10 days) foraging trips offshore within shelf seas, diving to the bottom to exploit benthic and demersal prey, and returning to coastal ‘haul-out’ sites^33,34^. Most research has focussed on adult movements and foraging strategies. Whilst some work has investigated foraging in grey seal juveniles (>12 months old) and young-of-the-year (YOY; 5 months old)^35,36^, and others have studied pup behaviour on and around the colony^37,38^, only Bennett *et al*.^24^ have examined the ontogeny of at-sea behaviour in recently-weaned pups across their first months of nutritional independence. Previous studies have demonstrated sex differences in the foraging behaviour of grey seal adults (seals of reproductive age)^32,36^, juveniles^36^, and YOY^35^. Sex differences in behaviour thus emerge from an early age^35^, but the timing of their onset is unknown. Development of diving and learning of successful foraging behaviour is also likely to be shaped by local experience, and the environment that pups encounter when they first go to sea. Oceanographic conditions and prey availability vary among regions, presenting different challenges for different subpopulations. Together, these factors may confer regional differences in the ontogeny of diving behaviour and thus the development of successful foraging strategies for grey seal pups.

**Figure 1:**
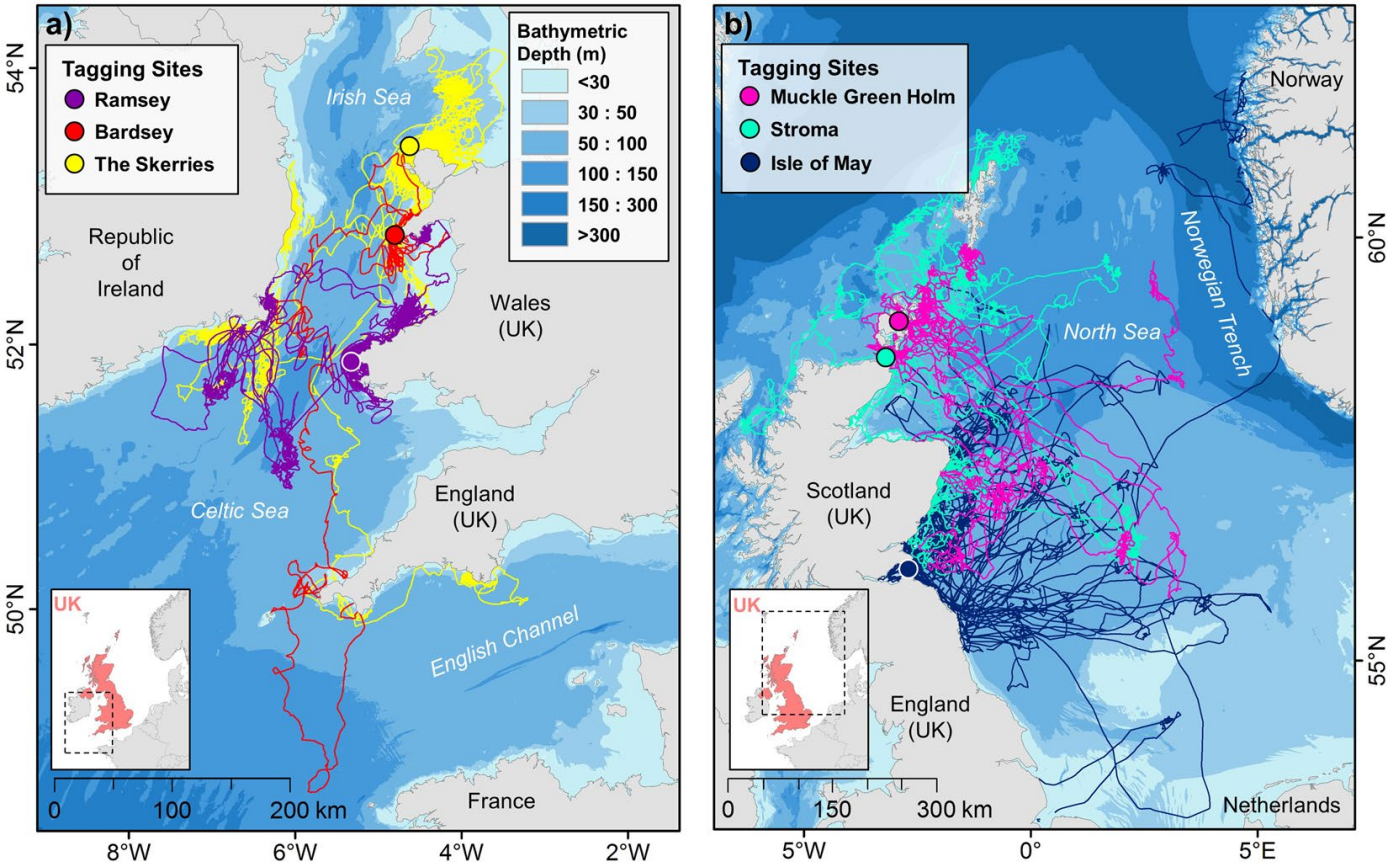
Pup tagging sites and trips at sea. Pups were tagged at six colonies in the United Kingdom (UK). Colonies were assigned to one of two geographic regions; (**a**) Celtic and Irish Seas (CIS), and (**b**) North Sea (NS). Tracks show pup trips (n = 836) during the initial four months after leaving the colony. Maps created in Esri ArcMap™ 10.2.2 (http://desktop.arcgis.com/en/arcmap/).

The United Kingdom (UK) is home to ~38% of the world grey seal population^39^ and has an obligation under European Union (EU) legislation to maintain this population in favourable conservation status (FCS)^40^. As part of this obligation, critical habitat must be identified for this species both on land and at sea to assess and mitigate anthropogenic disturbance. Current UK conservation management for grey seals at sea is largely based upon observations of adult movement^39^. Foraging behaviour has not yet been described for grey seal pups, however, given that they undergo profound physiological development during their initial months of independent life^24,25^, coupled with a need to explore their environment and develop knowledge of potential foraging areas, we should not expect their behaviour and habitat requirements to be the same as for adults. As pups develop diving skills, grow larger and acquire knowledge of their surroundings, we might expect that their behaviour begins to converge on that of adults, since adult behaviour represents successful foraging patterns. The main aim of this study, therefore, was not to quantify foraging in grey seal pups, but to investigate changes in at-sea behaviours relevant to the development of successful foraging skills during their first four months of independent life at sea. We used a unique, large (*n* = 52 individuals) animal-borne satellite and GSM telemetry dataset of location and dive (time-depth) data from recently-weaned pups born at six different colonies around the UK (Table 1). Ontogeny of foraging behaviour has been characterised in young seals by reductions in trip metrics (duration and distance), and increases in dive metrics (depth, duration, proportion of dives that are benthic, bottom time and proportion of day spent diving) with age^15,24,41,42^. Such changes in these metrics are indicative of an individual’s ability to maximise foraging opportunities within individual dives and/or over foraging trips, and are thus representative of greater foraging efficiency^15,24,41,42^. Thus, using generalized estimating equations in a generalized additive model framework (GEE-GAM), we investigated how these variables changed over time and compared the trajectories between the sexes and two distinct geographic regions (Celtic and Irish Seas (hereafter CIS) and North Sea (hereafter NS); Fig. 1). Furthermore, sexual segregation of foraging habitat may be manifested in the depth of water where males and females dive^35^. We therefore examined differences in the bathymetric depth of dive locations in the same way.

**Table 1:** Device deployment summary information.

Deployment site (year)	Region	Device type	No. tagged seals			Mean no. locations day^−1^ ± SD	Total no. trips	Total no. dives
			f	m	Total			
Isle of May (2001)	NS	SRDL	5	6	11	4.5 ± 2.3	109	N/A
Isle of May (2002)	NS	SRDL	5	5	10	5.2 ± 1.7	67	N/A
Bardsey (2009)	CIS	GPS-GSM	2	0	2	35.5 ± 5.4	23	3871
The Skerries (2009)	CIS	GPS-GSM	1	2	3	33.1 ± 5.7	141	9373
The Skerries (2010)	CIS	GPS-GSM	4	1	5	57.2 ± 13.8	212	46589
Ramsey (2010)	CIS	GPS-GSM	3	4	7	37.3 ± 9.7	162	27609
Muckle Green Holm (2010)	NS	GPS-GSM	4	3	7	22.5 ± 9.6	38	7417
Stroma (2010)	NS	GPS-GSM	5	2	7	24.4 ± 4	84	7941
		**Total:**	**29**	**23**	**52**		**836**	**102800**

Tagged pup sample sizes and tag duration by deployment site and year. Trip and dive numbers given are those included in the analysis after data cleaning and restriction to 120 days after leaving the colony. Although SRDL devices recorded dives, these could not be matched to bathymetric depth data and so were excluded from dive analysis. Colonies were assigned to two geographic regions; NS = North Sea, CIS = Celtic and Irish Seas.

## Results

### Trip behaviour

**Figure 2:**
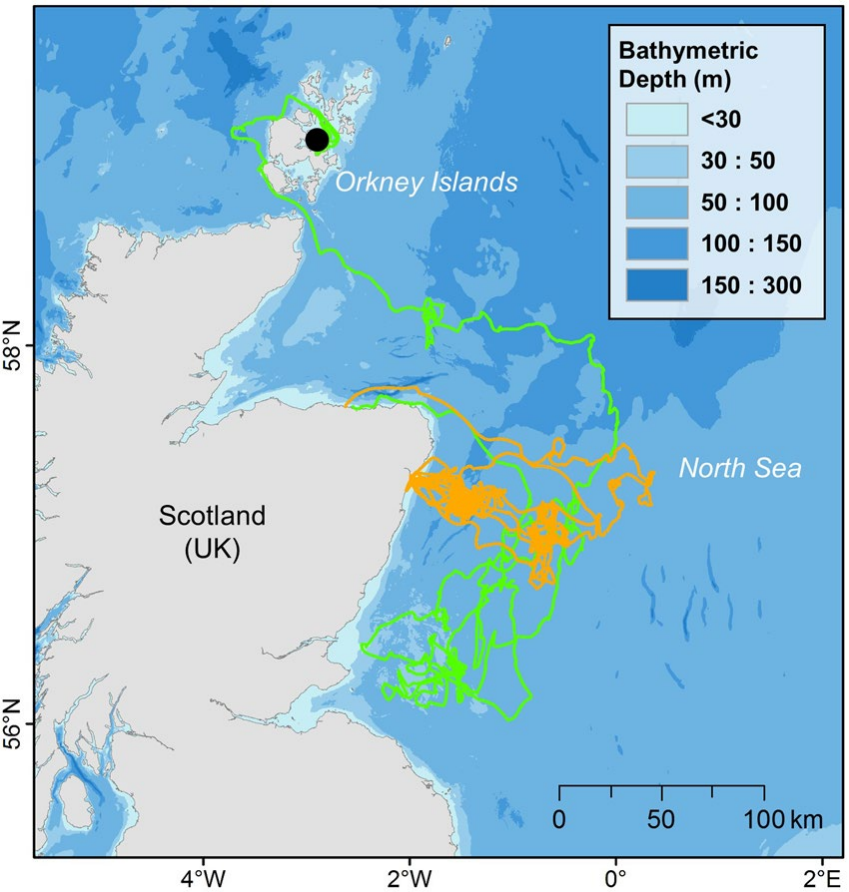
Changes in NS pup trip behaviour with time since departing the colony. Map shows initial exploratory trip of a pup from Muckle Green Holm, Orkney Isles (black dot), during which it did not haul-out for 64 days (green track). During the following 56 days, the pup performed repeated short-duration (5–14 days) foraging trips (gold tracks), travelling between the haul-out site and specific putative foraging areas. Map created in Esri ArcMap™ 10.2.2 (http://desktop.arcgis.com/en/arcmap/).

**Figure 3:**
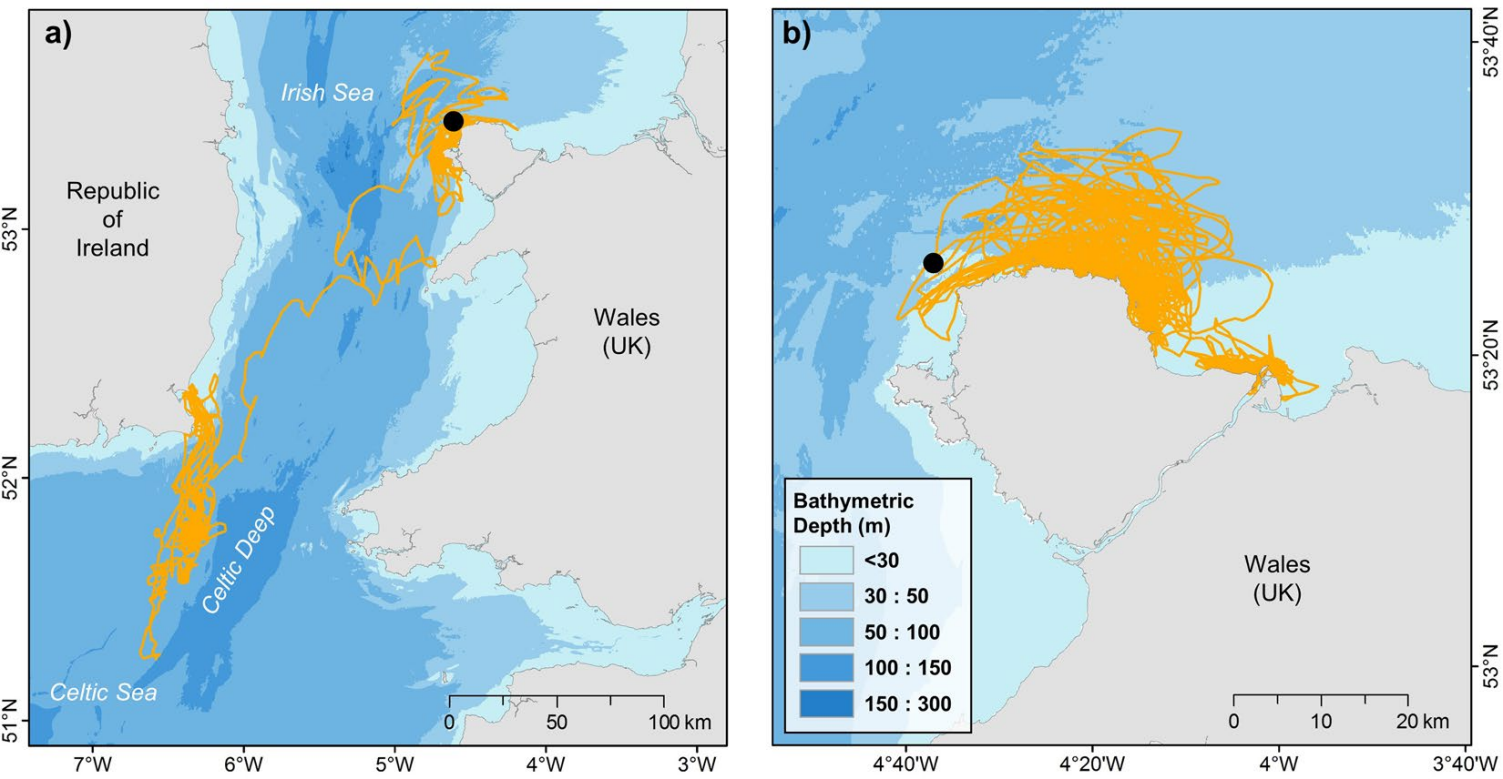
Differences in dispersal of CIS pups. Maps show trips of two pups from The Skerries over the first 4 months of independence. Pup (**a**) remains in areas adjacent to the colony (black dot) for ~50 days before dispersing to the southeast coast of Ireland, hauling-out at a popular grey seal haul-out site, and subsequently making repeated trips to putative foraging grounds on the edge of the Celtic Deep. Pup (**b**) remains in areas adjacent to the colony (black dot) for the entire 4 months. Maps created in Esri ArcMap™ 10.2.2 (http://desktop.arcgis.com/en/arcmap/).

All pups remained within the limits of the continental shelf, but NS individuals had a much wider dispersal pattern, and several pups travelled along the shelf break (Fig. 1). Although NS pups travelled far from their natal colonies on individual trips, all returned to haul-out locations on the east coast of Scotland and England. No pup crossed the shelf break into waters >200 m deep. However, one male from the Isle of May travelled between the UK and Norway on multiple occasions, diving to the bottom of the Norwegian Trench (Fig. 1b; >200 m). In general, pups from both regions explored new areas before settling into repeated trip behaviour, hauling-out in one or more locations and commuting back and forth to foraging grounds, as observed in adults^34^ (Fig. 2). Many NS pups undertook a prolonged exploratory phase shortly after leaving the colony, with 69% of pups (n = 24) spending >20 days offshore without returning to the coast, and some individuals exceeding 60 days offshore, which is substantially greater than typical trip durations seen in adults^34^. Only 18% of CIS pups (n = 3) performed a trip with duration >20 days. CIS pups remained much closer to land, generally dispersing along the coast of Wales and the Republic of Ireland (Fig. 1a). One female travelled south towards the north coast of France before returning to the south coast of England. Some individuals made repeated trips into the middle of the Celtic Sea, while others remained within 30 km of the coastline, and never travelled >50 km from their natal colony (Fig. 3).

**Figure 4:**
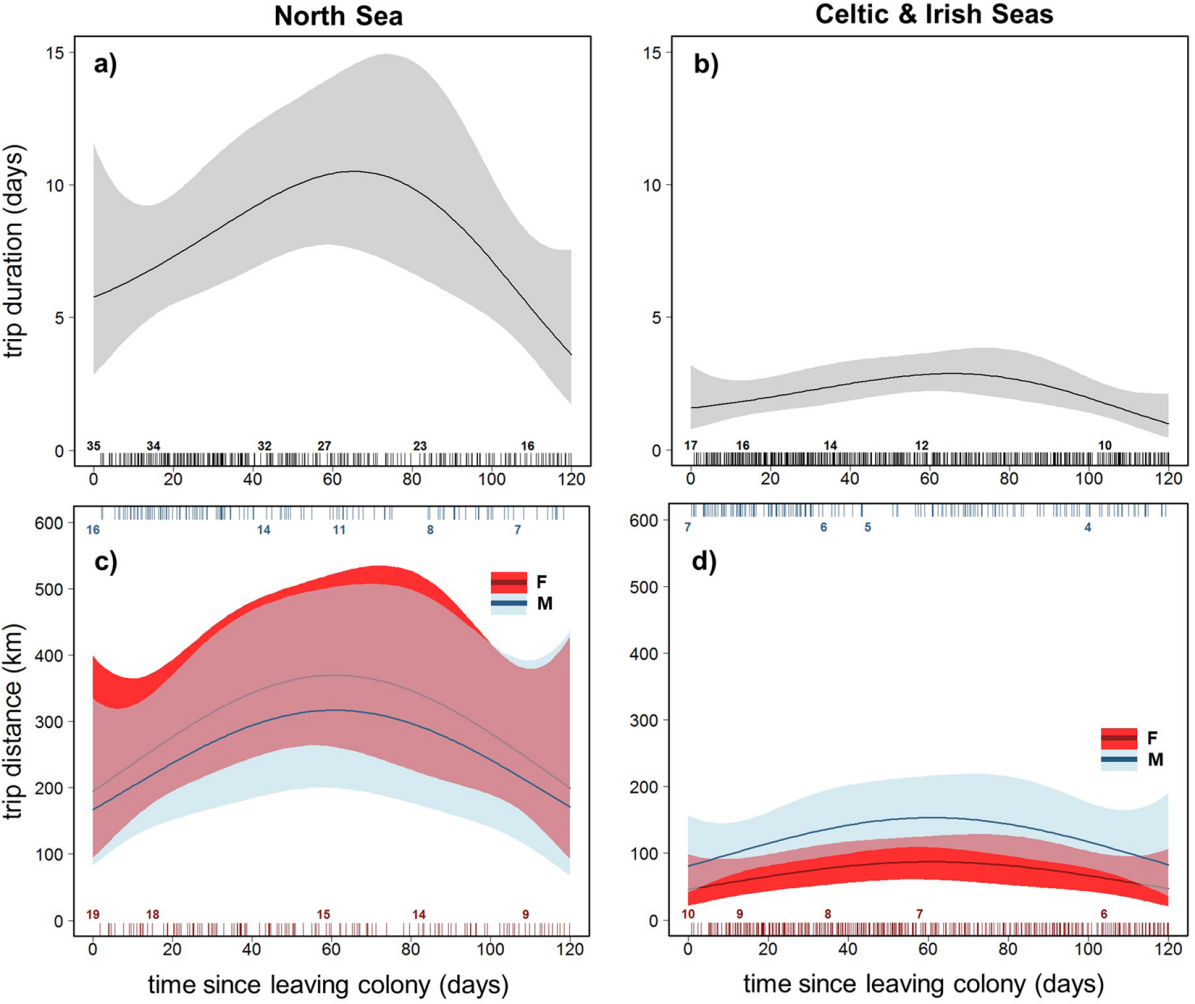
Sex and region differences in ontogeny of trip behaviour. Model-fitted values for trip duration (**a**,**b**) and trip distance (**c**,**d**) over time since leaving the colony. Solid lines show population mean responses by region (North Sea (NS) left, Celtic and Irish Seas (CIS) right), with associated GEE-based 95% confidence intervals (shaded areas). Pup trip behaviour changed significantly with time since departure. NS pups performed longer duration trips than CIS pups, however there was no sex difference (**a**,**b**). CIS males (**d**; blue) travelled further than females (red). Rug plots top and bottom show the distribution of data, colour-coded by sex, and associated numbers indicate pup sample size.

Trip duration increased significantly with time after leaving the colony for pups from both regions (Table 2; GEE-GAM; χ^2^_3_ = 15.2, *p* = 0.002), peaking at around 70 days before declining (Fig. 4a,b). However, trip duration was significantly longer for NS pups than CIS pups (Fig. 4a,b; GEE-GAM; χ^2^_1_ = 66.1, *p* < 0.001). There was no significant difference in trip duration between males and females in either region (GEE-GAM; χ^2^_1_ = 1.4, *p* = 0.233). Trip distance was also significantly affected by time since departure for pups from both regions (Table 2; GEE-GAM; χ^2^_3_ = 8.2, *p* = 0.042), peaking at around 70 days then declining (Fig. 4c,d). However, there was a significant effect of an interaction between region and sex on trip duration (Fig. 4d; GEE-GAM; χ^2^_1_ = 4.73, *p* = 0.03); NS pups travelled consistently further than CIS pups. CIS males travelled further than females, whilst there was no obvious sex difference in trip distance for NS pups.

**Table 2:** Model output.

Predictor Variables	Response Variables							
	Trips		Dives (daily means)					
	Duration (Figure 4a,b)	Distance (Figure 4c,d)	Max. Depth (Figure 5a,b)	Bathy. Depth (Figure 5c,d)	Prop. Benthic (Figure 5e,f)	Duration (Figure 6a,b)	Prop. Bottom Time (Figure 6c,d)	Prop. Time Diving (Figure 6e,f)
Time	χ^2^_3_ = 15.2, *p* = 0.002*	χ^2^_3_ = 8.2, *p* = 0.042*	—	—	—	—	—	—
Sex	χ^2^_1_ = 1.4, *p* = 0.233	—	—	—	χ^2^_1_ = 5.2, *p* = 0.023*	χ^2^_1_ = 2.5, *p* = 0.117	—	—
Region	χ^2^_1_ = 66.1, *p* < 0.001*	—	—	—	—	—	—	—
Time: Sex	χ^2^_3_ = 3.9, *p* = 0.268	χ^2^_3_ = 6.2, *p* = 0.1	—	—	χ^2^_3_ = 1.1, *p* = 0.774	χ^2^_3_ = 3.1, *p* = 0.369	χ^2^_3_ = 3.1, *p* = 0.378	χ^2^_3_ = 13.9, *p* = 0.003*
Time: Region	χ^2^_3_ = 4.1, *p* = 0.254	χ^2^_3_ = 3.3, *p* = 0.346	—	—	χ^2^_3_ = 13.1, *p* = 0.004*	χ^2^_3_ = 16.4, *p* < 0.001*	χ^2^_3_ = 14.9, *p* = 0.002*	χ^2^_3_ = 15, *p* = 0.002*
Region: Sex	χ^2^_1_ = 2.7, *p* = 0.099	χ^2^_1_ = 4.7, *p* = 0.03*	—	—	χ^2^_1_ = 0.1, *p* = 0.767	χ^2^_1_ = 0, *p* = 0.875	χ^2^_1_ = 9.3, *p* = 0.002*	χ^2^_1_ = 0.02, *p* = 0.885
Time: Region: Sex	χ^2^_3_ = 0.8, *p* = 0.852	χ^2^_3_ = 1.4, *p* = 0.708	χ^2^_3_ = 13.6, *p* = 0.003*	χ^2^_3_ = 10.4, *p* = 0.016*	χ^2^_3_ = 1.3, *p* = 0.74	χ^2^_3_ = 1.9, *p* = 0.591	χ^2^_3_ = 7.24, *p* = 0.065	χ^2^_3_ = 4.5, *p* = 0.215

Results of model simplification using backwards hypothesis testing with GEE-GAMs. Significant (*p* < 0.05) terms are shown with “*”. Interactions between variables are denoted by “:”. Where a variable was significant in an interaction, the significance of component interactions and/or individual fixed effects is not reported.

### Dive behaviour

**Figure 5:**
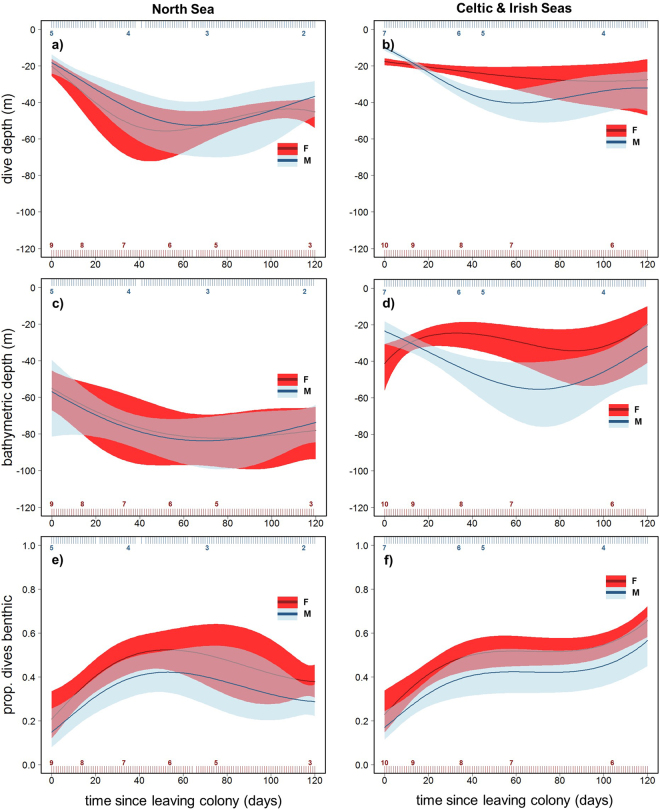
Sex and region differences in ontogeny of dive depth. Model-fitted values for daily mean maximum dive depth (**a**,**b**) and bathymetric depth of dive locations (**c**,**d**) over time since leaving the colony. Solid lines show population mean responses by region (North Sea (NS) left, Celtic and Irish Seas (CIS) right), with associated GEE-based 95% confidence intervals (shaded areas). Pups increased their dive depth rapidly over the initial 40 days (**a**,**b**), except for CIS females (**b**). NS pups dived in deeper water throughout (**c**). Sex differences in bathymetric depth of dive locations emerged from the outset in CIS pups, as females (red) dived in shallower areas (**d**). The proportion of dives that were benthic increased rapidly for all pups over the initial 40 days. However, females recorded marginally higher mean values than males in both regions (**e**,**f**). Rug plots top and bottom show the distribution of data, colour-coded by sex, and associated numbers indicate pup sample size.

A three-way interaction between time since departure, region and sex best explained variation in daily mean maximum dive depth (Table 2; GEE-GAM; χ^2^_3_ = 13.6, *p* = 0.003). Pups increased their dive depth rapidly over the first 40 days, except for CIS females, which showed a prolonged, more moderate increase (Fig. 5a,b). Sex differences in the change in dive depth over time were apparent in CIS pups, with males diving significantly deeper than females from 20–60 days after leaving the colony (Fig. 5b). The population mean maximum depth for CIS males during this period reached ~40 m, whilst females achieved ~25 m. Throughout the time series, NS pups dived significantly deeper than CIS pups, with both males and females reaching a maximum daily mean of ~50 m.

A three-way interaction between time since departure, region and sex best explained variation in daily mean bathymetric depth of dive locations (Table 2; GEE-GAM; χ^2^_3_ = 10.4, p = 0.016). NS pups and CIS males dived in increasingly deep water over the first 40 days after departure from the colony (Fig. 5c). CIS females remained in shallower water than males throughout the first four months at sea, averaging depths of ~30 m whilst mean bathymetric depth for male dives reached up to ~60 m (Fig. 5d). No significant sex difference was evident in bathymetric depth of dive locations for NS pups. Both male and female NS pups dived in significantly deeper water than CIS pups, reaching a maximum daily mean of ~80 m.

The daily mean proportion of dives that were benthic changed with time since departure, and the dynamic of this change was different between the regions (Table 2; GEE-GAM; χ^2^_3_ = 13.1, p = 0.004). Pups from both regions increased the proportion of benthic dives rapidly over the initial 40 days. This reached an asymptote for NS pups (Fig. 5e), but continued to increase for CIS pups (Fig. 5f). The trend showed some evidence of a decline in the latter half of the time series for NS pups, but confidence intervals were wide (Fig. 5e). Females performed a greater proportion of benthic dives than males throughout the time series in both regions (GEE-GAM; χ^2^_1_ = 5.2, p = 0.023). The daily mean proportion of benthic dives reached a peak at ~0.5 for NS females, ~0.6 for CIS females, ~0.4 for NS males, and ~0.5 for CIS males. Confidence intervals for the sexes overlapped in both regions. The effect of bathymetric depth on the proportion of dives that were benthic is presented in Supplementary Information (Supplementary Results: Effects of bathymetric depth on benthic diving).

**Figure 6:**
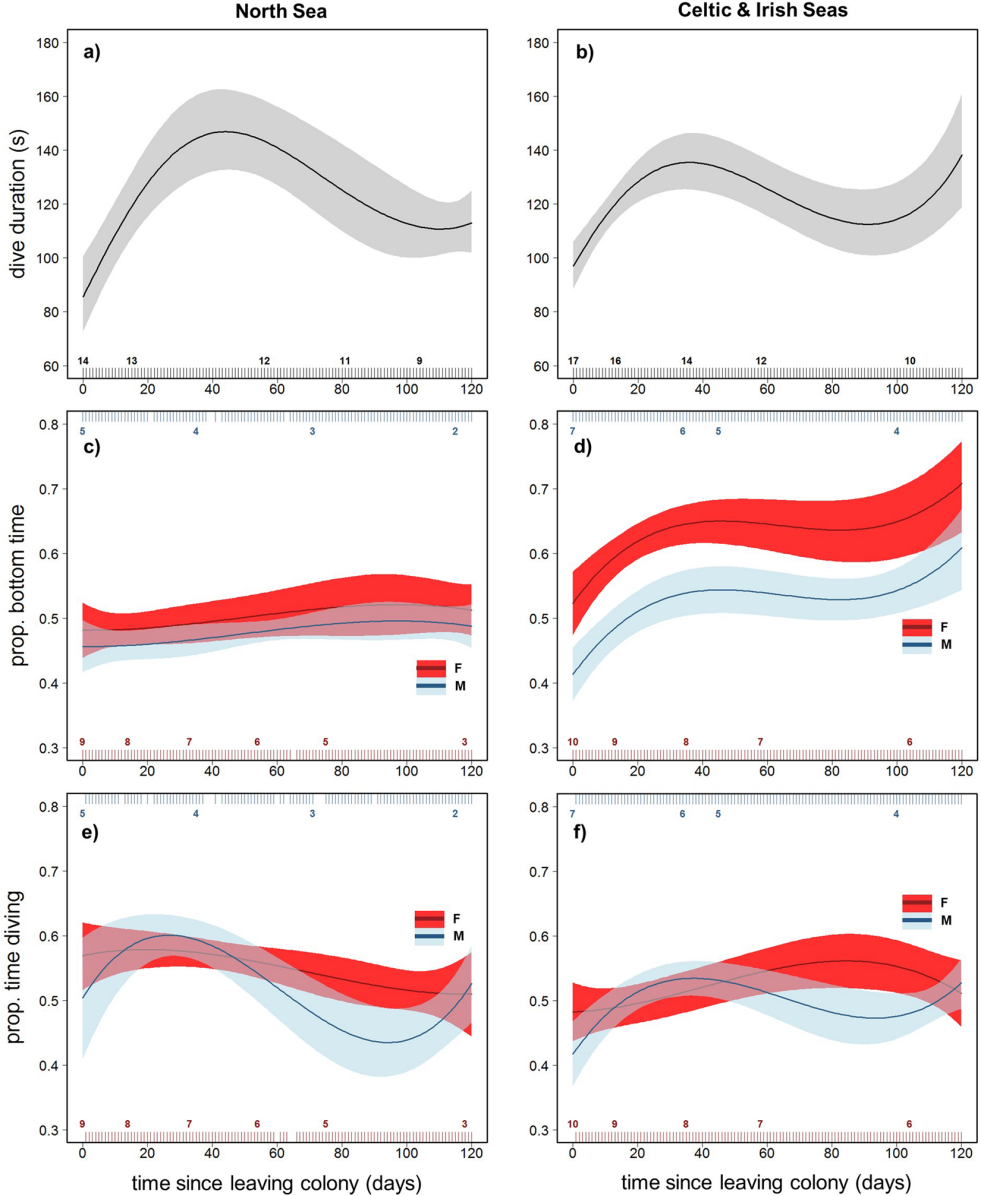
Sex and region differences in ontogeny of dive duration. Model-fitted values for daily mean total dive duration (**a**,**b**), bottom time (as proportion of total dive duration; **c**,**d**), and time spent diving (as proportion of 24 h period; e,f) over time since leaving the colony. Solid lines show population mean responses by region (North Sea (NS) left, Celtic and Irish Seas (CIS) right), with associated GEE-based 95% confidence intervals (shaded areas). Pups increased their dive duration rapidly over the initial 40 days (**a**,**b**), and there was no sex difference in dive duration. Females (red) had higher bottom time than males (blue) (**c**,**d**), although this was more marked in CIS pups (**d**). Females spent more time diving than males in the third month (**e**,f). Rug plots top and bottom show the distribution of data, colour-coded by sex, and associated numbers indicate pup sample size.

Daily mean dive duration was best explained by an interaction between time since departure and region (Table 2; GEE-GAM; χ^2^_3_ = 16.4, p < 0.001). There was no significant effect of sex on this metric (GEE-GAM; χ^2^_1_ = 2.5, p = 0.117). Similar to dive depth and bathymetric depth, pup dive duration increased rapidly over the initial 40 days at sea for both regions, before declining over the following 60 days (Fig. 6a,b). Peak mean dive duration for NS pups was marginally longer than for CIS pups (NS: ~140 s, CIS ~130 s).

Temporal patterns in bottom time differed between regions (Table 2; GEE-GAM; χ^2^_3_ = 14.9, p = 0.002). CIS pups showed a strong increase in bottom time over the initial 40 days at sea, before levelling off, then a further increase at ~100 days. NS pups showed a moderate increase over the whole time series, with bottom time remaining between 40–50% of dive duration (Fig. 6c,d). In addition, sex differences in bottom time differed between the regions (GEE-GAM; χ^2^_1_ = 9.3, p = 0.002). In both regions, females achieved higher bottom times than males (although 95% confidence intervals overlapped for NS pups; Fig. 6c). The difference between males and females was more pronounced in CIS pups: females achieved a maximum mean of ~70% of the dive spent in the bottom phase, whilst males achieved a maximum mean of ~55% (Fig. 6c,d).

Time spent diving per day varied significantly with time since departure, and the shape of this relationship was affected by sex (Table 2; GEE-GAM; χ^2^_3_ = 13.9, *p* = 0.003) and region (GEE-GAM; χ^2^_3_ = 15, *p* = 0.002). The sex difference was comparable between both regions (GEE-GAM; χ^2^_1_ = 0.02, *p* = 0.885). NS pups began diving ~14 h per day, then reduced time spent diving in the third month to ~10 h for males and ~12 h for females (Fig. 6e). CIS females initially spent ~11 h diving per day, which rose steadily to ~13 h in the third month (Fig. 6f). CIS males initially spent ~10 h per day diving, which rose steeply to ~13 h in the first month before declining back to ~11 h in the third month (Fig. 6f).

## Discussion

This study reveals that sexual segregation of behaviour can be exhibited as early as nutritional independence in capital breeders. Female pups from both regions spent more time diving per day than males. CIS females made shorter distance trips than males, diving in shallower water and achieving a higher proportion of the dive duration in the bottom phase. The same level of sexual segregation in depth, proportion bottom time and trip duration was not observed in NS pups, suggesting that sex differences in the ontogeny of foraging behaviour may be mediated by extrinsic factors. In both regions, pup behaviour changed rapidly: dive duration, depth, bottom time and benthic diving increased over the first 40 days after leaving the colony. These findings are important in the context of both foraging ecology and conservation management, as we outline below.

Grey seal adults exhibit substantial sexual size dimorphism^23^, which is thought to drive differences in feeding areas^32^. Grey seal pups are not size-dimorphic^24^, but seal pups and juveniles may experience differences in energy requirements before overt size and body composition differences emerge^43^. Kelso *et al*.^43^ found that male northern elephant seal (*M*. *angustirostris*) pups had higher rates of energy expenditure than females during the post-weaning fast, but were more effective at sparing protein reserves. These differences are likely related to the development of sex-specific metabolic strategies required for successful breeding^43^. Differences in metabolic demand during the ontogeny of foraging behaviour could therefore drive sex-specific feeding strategies and habitat requirements. Our findings support this possibility; we found that females from both regions spent longer performing behaviours consistent with foraging across two different temporal scales (individual dives and 24 h period). At the individual dive scale, time in the bottom phase is indicative of time at potential foraging depth, with the descent and ascent phases of the dive representing the transit to and from any potential prey patch^10^. Despite the lack of sex difference in total dive duration, females spent longer in the bottom phase than males relative to total dive duration. At the 24 h scale, females spent on average 2 h more diving than males in both regions. We also found a moderate sex difference in the proportion of dives that reached the seabed, with females performing more benthic dives than males. Females may therefore have increased chance of prey capture during individual dives, which could represent an energetic advantage^10,44^, and contribute to higher survival probability of female pups^29^. However, we cannot exclude the possibility that females spend more time diving because they are searching and are unsuccessful. Using direct observations of prey capture (i.e. stomach temperature telemetry, accelerometers or video cameras) to ground-truth putative foraging as identified from location and dive data would help to evaluate foraging success^45^, and draw links between differences in ontogeny of foraging behaviour and survival probability.

Sex differences in bottom time, proportion of benthic dives, and time spent diving per day may be related to differences in the type and quality of prey items consumed by male and female pups. For example, if females target lower energy prey items, they will need to spend longer foraging than males for the same energetic gain. Grey seals adults are benthic foragers with a broad diet that varies between the sexes^46,47^. Beck *et al*.^47^ used quantitative fatty acid analysis to investigate niche breadth in grey seals in the northwest Atlantic. They found that the diet composition of YOY animals was significantly broader than that of adults, but found no sex differences for young animals. However, grey seal diet varies regionally and seasonally^46,47^, and therefore extrinsic factors unique to certain locations may shape sex differences in diet for young animals. No specific information currently exists on the diet of recently-weaned grey seal pups in the UK once they have left the colony due to the logistical constraints of collecting tissue and/or faecal samples specifically from this age-class. However, a recent study of stable isotope ratios obtained from the teeth of older juvenile grey seals in the North Sea suggests that they feed on a wide variety of low trophic level, benthic prey close to shore^48^. The sharp increase in proportion of benthic dives over the first 40 days, and the subsequent reduction in trip distance, may therefore be indicative of pups learning to exploit benthic prey, and finding foraging grounds closer to shore where they can effectively reach the bottom. Additional dive analysis also suggests that shallow waters <20 m deep may represent important foraging habitat for grey seal pups (see Supplementary Results: Effects of bathymetric depth on benthic diving).

Water depth is an important regulating factor in foraging behaviour and habitat preference in older grey seals^49,50^. Breed *et al*.^35^ reported that adult and YOY females in the northwest Atlantic population forage in shallower water than males. Our data from CIS pups, showing that females dived in significantly shallower water than males, support these findings and suggest that water depth may play a key role in the development of habitat (and possibly diet) segregation among the sexes in some regions. We also found a moderate sex difference in trip distance for CIS pups, with males travelling further than females. Given that there was no sex difference in trip duration, this may mean that CIS males travel further offshore to forage compared to females, accessing deeper water, and potentially spending longer travelling per unit time spent foraging than females. CIS females performed a greater proportion of benthic dives in shallow water (<20 m) than males (see Supplementary Results: Effects of bathymetric depth on benthic diving). The fact that CIS pups dived in shallower water than NS pups likely means that they were able to achieve greater dive bottom time and proportion of benthic dives as they spent less time in the ascent and descent phases of the dive. Sex differences in trip distance and water depth of dive locations were not strongly evident for NS pups. As with other metrics, sex differences may be mediated by extrinsic factors that vary among regions, such as prey distribution, physical oceanography, and the diversity of available habitats. In general, the North Sea is a more homogeneous ecosystem, with less variation in bathymetry and habitat types than the Celtic and Irish Seas^51^, which may reduce sexual niche separation in NS pups.

Intra and inter-specific competition may impact trip distance and duration in central place foragers. Juvenile grey seals in the northwest Atlantic travel further and for longer on foraging trips than adults, likely as a result of competitive exclusion from the best foraging grounds closer to shore^52^. Age-related segregation has also been reported for other phocid species^53^. We found that NS pups travelled further offshore and performed longer trips than CIS individuals. Population density of grey seal adults is much higher on the east coast of Scotland compared to the Celtic and Irish Seas^39,54^. Moreover, Russell *et al*.^36^ showed that adult males in the North Sea reduce their time spent travelling to foraging locations in winter, whilst juveniles show an increase. Given that NS pups leave the colony during the winter months, and we see the longest trips performed during this time, competitive exclusion by conspecifics may be a feature of movement patterns specifically during the winter, forcing pups to make longer trips further offshore. In addition, harbour seals are present in coastal regions of the North Sea, but not in the Celtic and Irish Seas^54^. Inter-specific competition may also contribute to NS pups travelling further offshore than CIS pups.

Our results show that NS pups can make trips of over two months in duration, travelling greater distances than commonly observed in adult foraging trips and hauling out less frequently^34^. We also found that pups significantly reduced their trip duration and distance in the third month (Fig. 2). A similar temporal dynamic has been observed in other phocids, with young seals reducing trip duration after an initial increase^41^, and may be indicative of an increase in foraging efficiency, or a change in foraging strategy as pups age. Moreover, the higher initial trip duration and distance may represent an exploration phase in the development of NS pups. Votier *et al*.^55^ found that immature northern gannets (*Morus bassanus*) develop knowledge of foraging grounds during early-life exploratory trips. This may also be the case for grey seal pups, as, like gannets, they receive no parental guidance in the location of foraging resources. Furthermore, we found that some pups returned to forage repeatedly in areas that they had previously discovered during their initial exploratory trip (Fig. 2). Exploration may therefore be an important behaviour in determining future foraging success^8^.

CIS pups also performed exploratory trips, although their duration and distance was lower than those performed by NS pups. Individuals from NS colonies are not as geographically constrained as CIS pups by the proximity of land and shelf edge and therefore have more marine space to explore. Upon leaving the colony, CIS pups are more likely to encounter coastline, and therefore suitable haul-out locations, than pups in the North Sea. Alternatively, the offshore phase could be driven by environmental variables not measured in this study. For example, surface currents may direct pups further from land in the North Sea. The reduction in trip distance after 60 days for NS pups may therefore be related to a seasonal change in physical oceanography, or an increase in their ability to resist surface currents as muscle strength improves. Grey seals are known to rest at sea^36^, and this study provides further evidence that they do not need to return to shore to rest, even when very young.

Our results show that pup movements can change rapidly throughout the initial months at sea. Therefore, accurately quantifying foraging effort from these data may require extension of current analytical techniques, such as state-space models (SSMs)^45^, to account for temporal changes in movement patterns. Moreover, as a priority for future work, analysis of pup foraging habitat preference may allow us to infer potential prey species based on habitat features such as substrate type, and further assess the implications of early-life sexual segregation in movement patterns for foraging ecology.

In addition to ontogenetic changes in muscular and cardio-vascular systems, oxygen storage capacity and metabolic rate, and the development of knowledge of profitable foraging areas, there are likely to be seasonal changes in foraging habitat and prey distribution which may further explain differences in pup behaviour over time. Given that pups leave the colony on different dates in both regions (see Supplementary Note: Colony departure dates), local conditions may dictate some of the patterns observed here. Bennett *et al*.^56^ have shown that maximum dive depth of adult southern elephant seals may be regulated by seasonally-mediated factors, however, due to a paucity of tracking data from post-breeding adult grey seals in the UK, seasonal changes in at-sea behaviour are unclear. It was therefore not possible to disentangle ontogeny from seasonal effects on pup behaviour. Furthermore, some of the variance in early-life behavioural ontogeny may be explained by the fact that post-weaning fast duration varies among individuals^24^, and age at the point of departure from the colony is not equal for all pups. Natal and weaning dates were not known for all pups in this study, and time since departing colony was therefore used as a measure of at-sea experience. Future research should aim to achieve simultaneous tagging of adults, juveniles and pups, coupled with colony-based monitoring, which will allow us to further tease apart intrinsic and extrinsic drivers of variation in grey seal foraging behaviour and investigate the potential for competitive exclusion^36^.

Investigating the factors that affect the ontogeny of early-life behaviours is key to understanding how populations may respond to natural and anthropogenic threats. Bennett *et al*.^22^ suggested that grey seal pups have an average of 36 days in which to find food after leaving the colony before their protein reserves are critically depleted and starvation occurs. Our results show that profound changes in pup behaviour happen during the first 40 days after departure from the colony, indicating this initial period at sea is likely of particular importance for development of effective foraging strategies. Consequently, pups may be most vulnerable to disturbance from a number of growing anthropogenic activities, such as increased vessel traffic^57^, intensive fishing practices^58^ and offshore construction^59^ during this period, with substantial consequences for survival. Given the importance of early-life survival for maintaining stable populations^31^, and the rapid development of key behaviours during this period, conservation managers should make special considerations for pups during their initial months at sea to effectively mitigate these threats and avoid population-level impacts. With continuing development of biologging technology and analytical techniques, further work is urgently needed to fully explore and describe the ontogeny of fundamental behaviours in naïve marine predators and identify critical habitat for young animals during their most vulnerable life stage.

## Methods

### Instrumentation

Two different telemetry device models were deployed on 52 recently-weaned grey seal pups at six UK breeding sites in 2001 and 2002^24^, and in 2009 and 2010^60^ (Table 1). Earlier deployments (2001–2002; n = 21) were Argos Satellite Relay Data Loggers (SRDL; Sea Mammal Research Unit, UK), and later deployments (n = 31) were Fastloc® GPS-GSM tags (GPS phone tags; Sea Mammal Research Unit, UK). Individuals were captured post-weaning for device application. When anaesthesia was required (due to additional procedures not related to this study; CIS 2010 and all Isle of May deployments), pups were administered with 0.025 mg kg^−1^ intravenous Zoletil_100_® (Virbac, France)^24,60^. Following McConnell *et al*.^34^, a tag was glued to cleaned, dried fur at the base of the skull using RS Quick-Set Epoxy Adhesive (RS Components Ltd., UK; 2001–2009), or Loctite® 422™ cyanoacrylate adhesive (Henkel, UK; 2010). All experimental protocols were carried out with UK Home Office approval under project licences #60/2589 (2001–2002), and #60/4009 (2009–2010), in accordance with the Animals (Scientific Procedures) Act 1986. In total, 7057 days of data were recorded from 52 pups (for information on tag duration see Supplementary Note: Tag duration).

#### Horizontal movement data

Whilst both SRDL and GPS-GSM devices transmitted location data at irregular intervals, mean number of location fixes achieved per day was much higher for GPS-GSM tags (Table 1). Argos-derived location estimates from SRDLs also carry a greater spatial error, ranging from 50 m to >2.5 km^61^. Erroneous Argos location observations were eliminated using the standard technique of filtering with a maximum speed threshold of 2 ms^−1^ ^62^. Remaining locations were then processed with a Kalman filter to improve location accuracy^54^. Kalman filter observation model parameters were taken from Vincent *et al*.^61^, and process model parameters were based on average speeds of 142 seal GPS tracks^54^. Erroneous GPS locations were identified and excluded using residual error thresholds and number of satellites^36^.

Devices also recorded dive and haul-out data derived from integrated conductivity and pressure sensors. Following Russell *et al*.^36^, a seal’s location during a haul-out event was taken as the mean of all latitude and longitude estimates during the time hauled-out. If no location estimates were recorded during the haul-out interval, the location was derived using linear interpolation to a midpoint between the pre and post observed location fixes. Interpolated haul-out locations were flagged as unreliable if there was no adjacent observed location within 6 h. The location data were then restricted to discrete ‘trips’ between haul-out events. Trips were only included in the analysis if they had a reliable haul-out location on land at both the beginning and end. One individual hauled-out repeatedly on an offshore oil rig in the central North Sea >250 km from land; these haul-outs were classed as on land and the associated trips were included in the analysis. Seals often wait in the water between haul-out events for tidal sites to become available, when they may sleep either on the seabed or at the surface^63^. To exclude this behaviour, as it is not relevant to foraging, trips <8 hr in duration and with a maximum distance <500 m from the coast were also omitted from the analysis^64^. Finally, as tag duration varied between individuals (from 13 to 337 days; see Supplementary Note: Tag duration), data were clipped at 120 days after leaving the colony to ensure a robust sample size throughout the time series for statistical analysis^65^. Sample sizes are presented alongside rug plots (Fig. 4). The resulting dataset comprised location and haul-out data from 52 individuals; 23 males and 29 females (Table 1; Fig. 1; 836 trips). The duration and total distance of each trip was calculated alongside days since first leaving the natal colony at the mid-point of the trip. Total distance was calculated as the sum of all step lengths between successive location fixes during a trip, regularised to 30 min intervals. Days since leaving colony was used to give a measure of the at-sea experience of the pup.

#### Dive data

GPS-GSM tags classified dives as periods when the pressure sensor recorded depths >1.5 m for >8 s. These devices recorded depth readings at 4 s intervals throughout a dive, which were then abstracted to 11 inflection points by an algorithm onboard the device before data transmission^66^. Although SRDLs also recorded dive data, tag parametrisation was different to that of GPS-GSM tags (SRDLs only recorded dives >6 m depth with four inflection points). Furthermore, the lower frequency of successful transmissions and higher spatial error of concomitant Argos-derived location estimates meant that SRDL dive data could not be accurately matched to a location, and thus to bathymetric depth, and were therefore excluded from all dive analyses. For GPS-GSM dive data, the maximum dive depth and total dive duration were extracted for each dive. A dive was treated as any time below the depth threshold (1.5 m). Devices also transmitted two-hourly summaries of data, detailing the proportion of time the device was in either “haul-out”, “dive” or “cruise” (device is wet and above 1.5 m) mode. These data were used to calculate the total number of hours spent diving per individual per day. Only days with data for all twelve summary intervals were used.

To investigate changes in the proportion of benthic dives performed by pups, and the bathymetric depth of dive locations, dives were first matched to adjacent location fixes in time using the mid-point between dive start and end times. The location for each dive mid-point was then calculated using linear interpolation between prior and post location fixes. Interpolated dive locations with no adjacent observed location fix within 15 min could not be accurately matched with bathymetric depth data and were therefore excluded from the analysis. Bathymetric depth was extracted for each dive location from the harmonised 1/8 arc minute * 1/8 arc minute (~230 m) gridded Digital Terrain Model (DTM) for European Waters which is freely-available through the European Marine Observation and Data Network (EMODnet) Portal for Bathymetry^67^. Benthic dives were classified following Ramasco *et al*.^68^, using a mixture distribution model approach (see Supplementary Methods: Classification of benthic dives). The bathymetric depth range of the study area is shown in Fig. 1. After filtering, a number of dives (15%) recorded a null or positive bathymetric depth value, due to interpolated dive locations falling too close to the coast, and were subsequently removed. As with trip data, the resulting dive dataset was clipped to 120 days after leaving the colony to ensure a robust sample size throughout the time series. Sample sizes are presented alongside rug plots (Figs 5 and 6). Lastly, as seals may perform successive shallow dives while resting close to haul-outs, and this is not related to foraging behaviour, any dive with a maximum depth <5 m was excluded. The final dataset comprised 102,800 dives from 31 individuals (Table 1).

### Statistical analysis

Trip (duration and distance) and dive metrics (depth, bathymetric depth of dive locations, proportion of dives that were benthic, duration, bottom time and proportion of day spent diving) were analysed using generalised estimating equations within a generalized additive model framework (GEE-GAMs) using the “geepack” and “splines” packages^69^ in R^70^. The GAM approach allows the inclusion of smoothed terms to investigate non-linear relationships^71^. However, GAMs are not robust to the serial autocorrelation within individuals that is inherent in longitudinal telemetry datasets. GAMs can be extended to include autocorrelation structures and random effects, however the GEE approach allows the inclusion of an unstructured correlation coefficient, which is more appropriate for telemetry data as it estimates all correlations between within-individual observations independently^72^. Furthermore, this method allows the prediction of population mean responses by averaging across individuals. This approach has been previously applied to study temporal movement trends in seal telemetry datasets^36^.

We investigated ontogeny in pup dive behaviour using a number of metrics, at a temporal resolution of one day by calculating daily means per individual per day. As pups grow, their muscular and cardio-vascular systems develop, and they convert blubber into lean mass, becoming less buoyant^25,73^. Their ability to dive to, and remain at depth should therefore increase over time^24^. Daily mean dive maximum depth and duration were used to track changes in diving ability over time. For air-breathing benthic foragers, the depth of water in which dives occur is also relevant to their ability to dive to and remain at foraging depth. The bathymetric depth of water where dives occurred was also modelled in the same way. Optimal diving theory (ODT) suggests that benthic foragers will maximise time at the seabed (and therefore probability of successful foraging), and minimise time spent in the ascent and descent phases of a dive and at the surface^74^. We therefore investigated changes in the proportion of dives that were benthic, and in dive bottom time (the proportion of a dive’s duration spent at >80% of the maximum dive depth; a measure of time spent at foraging depth relative to descent and ascent phases of a dive)^75^. Lastly, pups may maximise time spent underwater (and therefore foraging opportunities) over bouts of short dives, rather than individual long dives^10,44^. We therefore investigated changes in the mean proportion of time spent diving per individual per day (24 h period).

Pup behaviour may change through time, and the dynamics of this change may differ between the sexes, and/or geographic regions (due to differences in habitat features such as coastal geography, prey availability and bathymetry). Therefore, response variables were analysed in separate models as a function of time since leaving colony (days; as a smoothed term), sex (as a categorical term) and region (as a categorical term) in a three-way interaction. Model selection was performed by backwards hypothesis testing from GEE-based p-values until arriving at a minimum adequate model. Colonies were assigned to one of two geographic regions (Table 1; North Sea or Celtic and Irish Seas). There was considerable spatial overlap of areas used by pups from colonies within each of the two wider geographic regions (Fig. 1), such that region rather than colony was used in the models for the sake of parsimony, and to maximise statistical power. 95% confidence intervals around model-predicted means were calculated by parametric bootstrapping using GEE-based uncertainty parameters^54^. Scale-corrected Pearson’s residuals were checked for normal distribution by visual inspection in all models. For models with continuous response variables (all except bottom time, benthic diving and proportion of day spent diving), Gamma and Poisson error structures were considered to improve normality, but in all cases a Gaussian error structure with log-link function proved superior. Bottom time, proportion of dives that were benthic and proportion of day spent diving (proportion data) were modelled with a binomial error structure with logit-link function.

### Data availability

The datasets used in the current study are available from DJFR; dr60@st-andrews.ac.uk.

## Electronic supplementary material

Supplementary Information
