# Supplementary material for: Intrinsic and extrinsic factors drive ontogeny of early-life at-sea behaviour in a marine top predator
Source: Sci Rep. 2017 Nov 14;7:15505. doi: 10.1038/s41598-017-15859-8 (PMC5686064; doi:10.1038/s41598-017-15859-8)
Supplement: Supplementary file 1 — Supplementary Information [file 41598_2017_15859_MOESM1_ESM.pdf]

Supplementary Information for:

Intrinsic and extrinsic factors drive ontogeny of early-life at-sea behaviour in a marine top predator

Matt I. D. Carter*; Deborah J. F. Russell; Clare B. Embling; Clint J. Blight; David Thompson; Philip J. Hosegood; Kimberley A. Bennett

Scientific Reports (2017)

*matthew.carter@plymouth.ac.uk

Supplementary Results: Effects of bathymetric depth of benthic diving

To investigate how the temporal changes in proportion of benthic dives were related to the bathymetric depth of water where those dives occurred, we modelled the daily proportion of dives that were benthic in a GEE-GAM as a function of a four-way interaction between time since departing colony (days; as a smoothed term), bathymetric depth (m; as a smoothed term), region (as a factor) and sex (as a factor). Bathymetric depth was taken as the mean of dive bathymetric depth values per pup per day. Individual was used as the grouping variable to generate a population mean response. Models were weighted by the number of dives in each time (day) – depth (m) category.

The daily proportion of benthic dives was best explained by a three-way interaction between time since departure, sex, and bathymetric depth (Supp. Fig. S1; $\chi^2_9 = 56.5$, $p < 0.001$) and a three-way interaction between time since departure, region and bathymetric depth (Supp. Fig. S1; $\chi^2_9 = 49.6$, $p < 0.001$).

All pups increased the depth at which they were able to perform benthic dives over the initial 40 days (Supp. Fig. S1). Although confidence intervals were wide, due to the model predicting across time and space in which few dives occurred, it is evident that the depth band between 60-80 m becomes important for benthic diving in all pups from 60 days onwards. Shallow waters (<20 m) appear to be important for benthic diving in NS pups during the first 10 days, then again later from 60 days onwards as pups perform shorter distance trips and remain closer to the coast. This supports the findings of Hanson *et al.*[1] that NS juveniles forage on benthic prey in nearshore habitat. CIS females performed a greater proportion of benthic dives in shallower water (<20 m) than CIS males throughout the entire time series. Although results presented in the main article show that the mean water depth of dives for CIS females remained shallow throughout the time series, results presented in Supp. Fig. S1 show that some individuals likely entered deeper water towards the end of the time series and were able to reach the bottom.

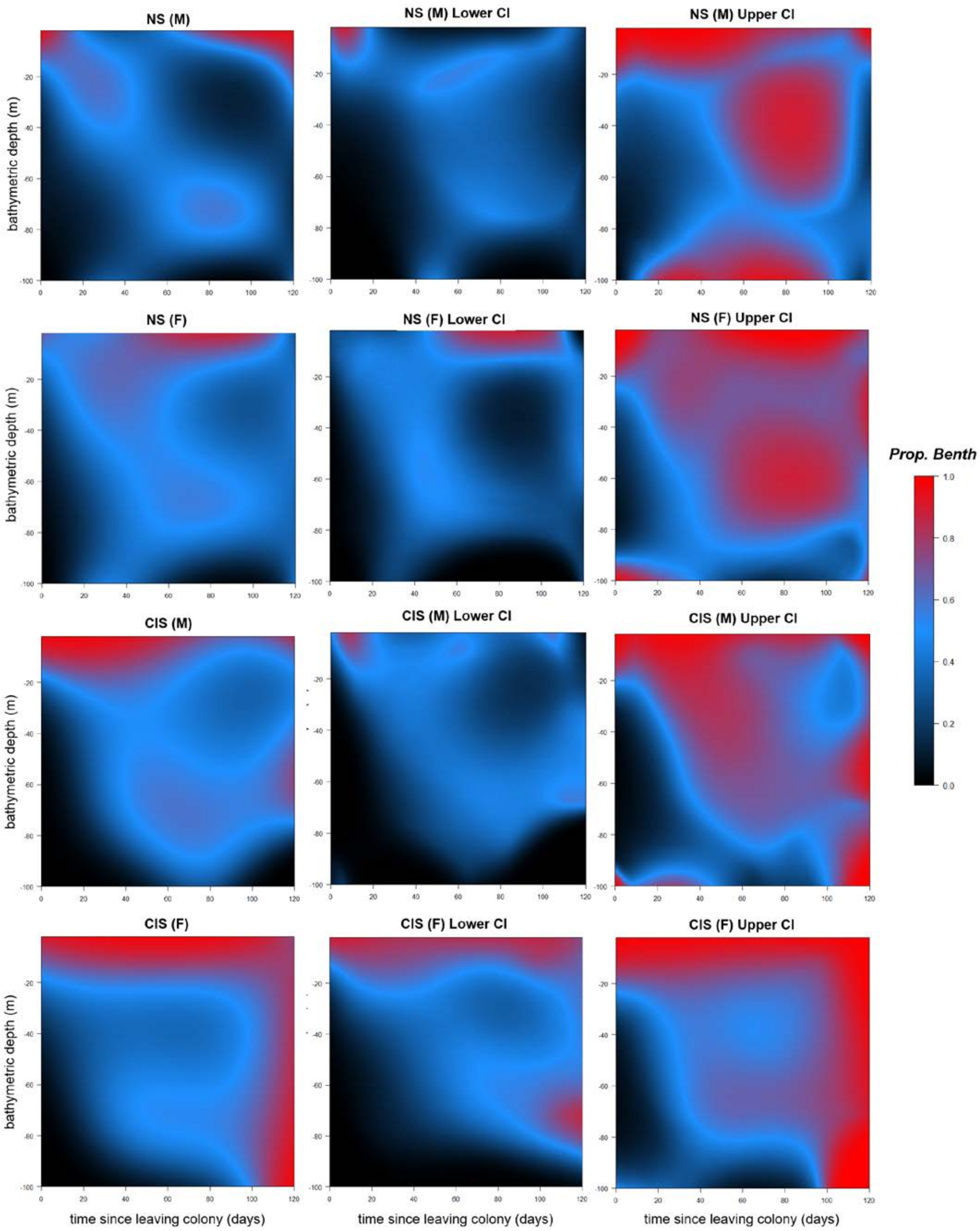

Supplementary Figure S1: Effects of bathymetric depth on benthic diving. Surface plots show model-predicted population mean estimates for the proportion of dives that were benthic (colour palette) by bathymetric depth (y axis) and time since leaving colony (x axis). Left hand panels show model-predicted population mean estimates, centre panels are GEE-based lower 95% confidence intervals, and right-hand panels are upper 95% confidence intervals.

Supplementary Note: Colony departure dates

It is possible that pups born later in the breeding season undergo a shorter post-weaning fast on land, and therefore depart the colony at a younger age than those born earlier due to some seasonal effect such as weather patterns. It is also possible that pups that leave later experience different water column characteristics and food availability than those that leave earlier. These factors may have an effect on subsequent behavioural development. For the majority of pups included in this study, natal and weaning dates were not observed, and so calendar age at point of departure from the colony was not known. However, for 17 individuals instrumented on the Isle of May, age at weaning and departure and calendar date of departure are all known. We therefore tested whether natal date had an effect on post-weaning fast duration using a Spearman's Rank correlation test. There was no significant correlation between natal date and post-weaning fast duration (Supp. Fig. S2; Spearman's Rank Correlation, $r = -0.079$, $n = 17$, $p = 0.763$).

Earliest and latest departure dates are presented for each deployment site in Supp. Table S1. Calendar date of departure differed by 73 days across the whole study, mostly as a result of the wide spread of departure dates in 2010. There was a mean of 40 and 58.5 day difference in calendar day of earliest and latest departure between NS and CIS colonies respectively, which reflects the shift in timing of the breeding seasons in different parts of the UK. The Isle of May calendar departure days differed by approximately 1 month between the earliest and latest. All departure dates within any of the other deployments were within 16 days of each other, which should minimise any effect of calendar day of departure on behaviours measured.

Supplementary Table S1: Pup departure dates. Table shows day of the year (DOY) of earliest and latest departure from each of the study sites in each sample year.

Deployment site (year)	Region	Device type	DOY earliest departure	DOY latest departure	Max. difference (days)	No. individuals
**Isle of May (2001)**	NS	SRDL	338	366	28	11
**Isle of May (2002)**	NS	SRDL	333	357	24	10
**Bardsey (2009)**	CIS	GPS-GSM	296	303	7	2
**The Skerries (2009)**	CIS	GPS-GSM	295	302	7	3
**The Skerries (2010)**	CIS	GPS-GSM	300	311	11	5
**Ramsey (2010)**	CIS	GPS-GSM	293	299	6	7
**Muckle Green Holm (2010)**	NS	GPS-GSM	346	361	15	7
**Stroma (2010)**	NS	GPS-GSM	349	365	16	7

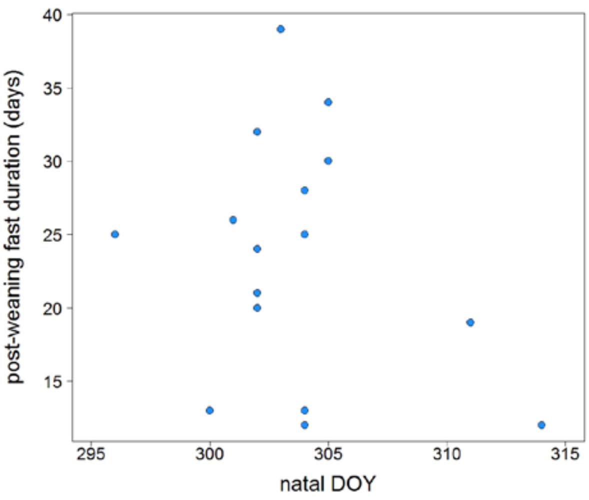

Supplementary Figure S2: Effect of natal date on post-weaning fast duration. Pups born on a later day of the year (DOY) do not have a shorter post-weaning fast than those born earlier.

Supplementary Note: Tag duration

Transmission duration ranged from 13 to 337 days (mean 177 days ± 81.1 days SD). Tags ceased to transmit in a relatively linear fashion over time (Supplementary Fig. S3). Battery performance will vary between devices depending on tag parametrisation; i.e. higher duty cycles and frequency of transmission attempts require higher battery demand. In this study, GPS-GSM devices recorded and transmitted more data than SRDLs. However, the longevity of the two device types was comparable (Supplementary Fig. S3). This is likely due to an increase in battery efficiency and power demands in the newer GPS-GSM devices. Tag failure due to battery exhaustion is likely to occur from around 120 days after deployment.

Both Argos SRDLs and GPS-GSM devices record location and dive data, however data transmission methods differ. Argos data are relayed via polar-orbiting satellites, whilst GPS-GSM devices transmit data via the mobile phone network once a tagged individual comes into GSM coverage. Therefore, it is possible that some of the GSM devices continued to collect data past the recorded maximum tag duration, but that these data were not transmitted as the seals did not enter GSM range before the battery expired.

A General Linear Model (GLM) was fit to investigate differences in tag duration as a function of sex, region and year of deployment. There was no significant difference in tag duration between the sexes (GLM; $F_{49,50} = 16.26$, $p = 0.961$), between the regions (GLM; $F_{50,51} = 0.93$, $p = 0.341$), or among deployment years (GLM; $F_{48,49} = 0.04$, $p = 0.837$). Although it was not possible to accurately assess pup survival for the above reasons, pup mortality is likely to be a feature of the observed trend in tag duration during the first six months as individuals are at greater risk of starvation and death[2].

Three of the pups tagged in Wales were later re-encountered. One female was found dead ashore 13 days after leaving Bardsey. A necropsy did not reveal any obvious cause of death[3]. Another female from Bardsey was found ashore in the south-west of England in an emaciated condition, two months after leaving the colony, and was taken to the National Seal Sanctuary for rehabilitation. A male from The Skerries was by-caught and died off the south-east coast of Ireland around six months after leaving the colony. The fate of all other pups is unknown.

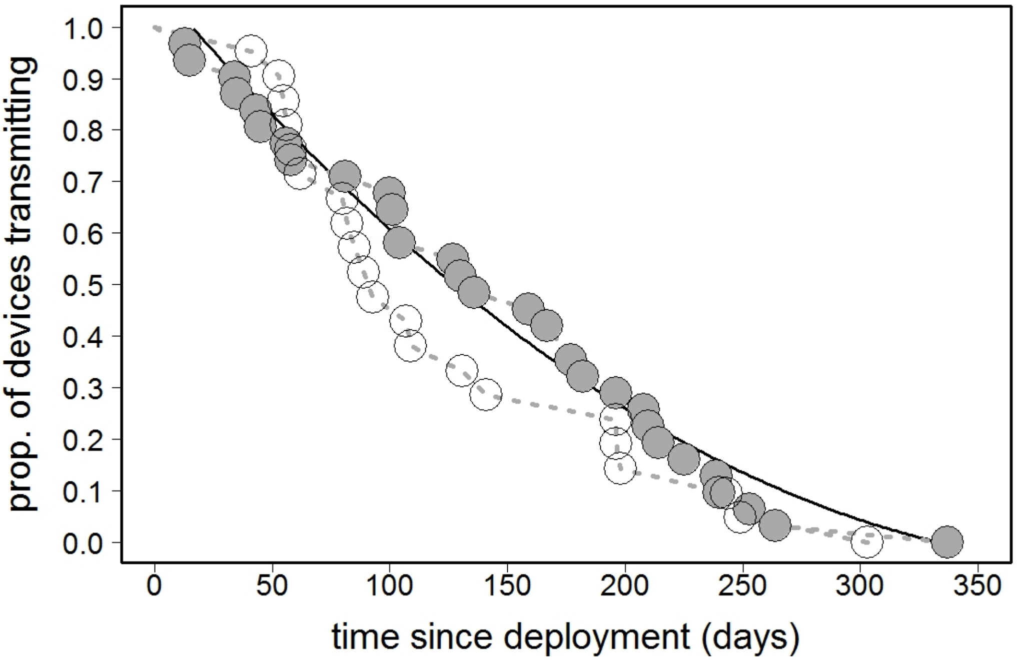

Supplementary Figure S3: Tag duration by device type. Time-series plot shows the proportion of SRDL (open circles; n=21) and GPS-GSM (filled circles; n=31) devices transmitting as a function of time since deployment. Points indicate when a device stops transmitting. Black line is a second order polynomial regression curve for all deployments (n=52).

Supplementary Methods: Classification of benthic dives

We first calculated the difference, for each dive, between the maximum dive depth and the bathymetric depth of water where that dive occurred (hereafter referred to as "distance from seabed"). Many distance values were negative, indicating that the seal dived deeper than the water column depth. This is likely due to differences in the way that both forms of depth data are recorded. The bathymetric depth data used here are mostly supplied as estimates of water depth at Lowest Astronomical Tide (LAT), whereas dive depths are recorded as depth below the surface regardless of tidal state. Given that seals dive at all states of tide, the resulting dive depth values may exceed the estimated water column depth. Furthermore, dive locations were assigned using interpolation between GPS location fixes (see main article), and this may lead to uncertainty in locations, affecting the accuracy with which dives can be matched to bathymetric data. To overcome this uncertainty, benthic dives may be identified using a mixture distribution model approach[4].

Following Ramasco *et al.*[4], we fit a mixture of normal distributions to the frequency distribution of distances from seabed (Supp. Fig. S4). We decided on three distributions *a priori* as we expect that the data contain benthic, midwater and surface (transit/travelling) dives. We then took the distribution with mean closest to 0 (-3.69 m) as representing the distribution of distance from seabed for benthic dives[4]. Following Ramasco *et al.*[4], the threshold for benthic dives was taken as the upper 95th percentile of this distribution. Benthic dives were therefore determined as any dive with a distance to seabed ≤ 0.31 m (Supp. Fig. 4).

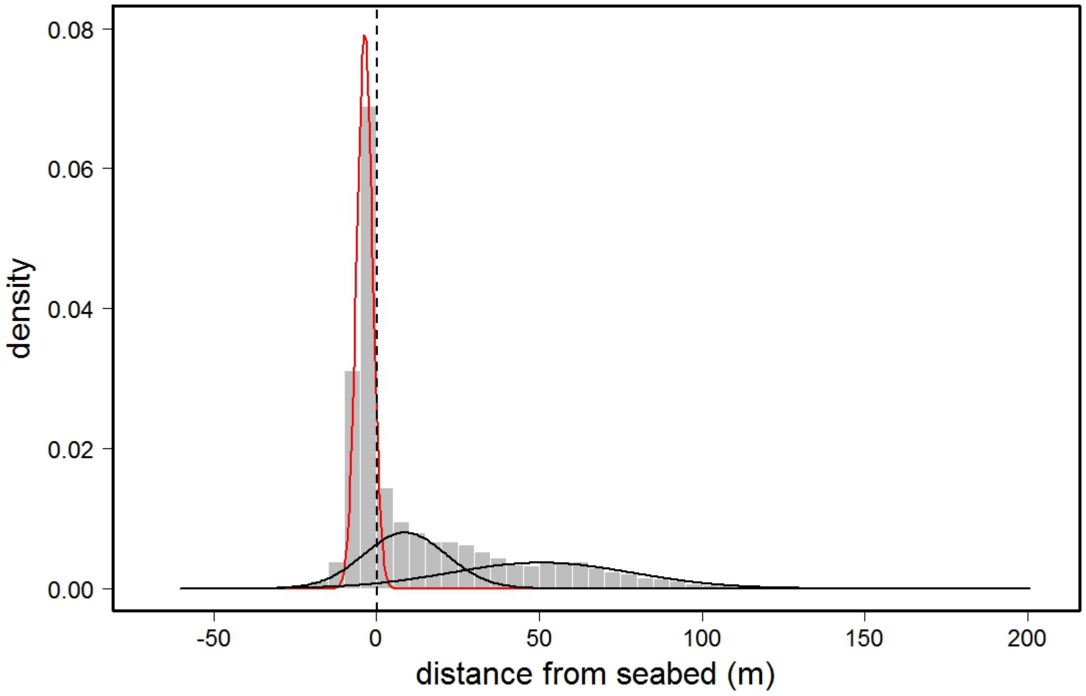

Supplementary Figure S4: Classification of benthic dives. Histogram shows the density distribution of distance from seabed for all dives. A mixture of three normal distributions was fit to the data (solid lines). The distribution with mean closest to 0 (-3.69 m; shown in red) was taken to be the distribution of distance from seabed for benthic dives. The upper threshold for classification of benthic dives was set at the upper 95th percentile of this distribution (0.31 m; dashed line).

References for Supporting Information

1. Hanson, N., Jones, E. L. & Harris, R. N. Multi-decadal and ontogenetic trophic shifts inferred from stable isotope ratios of pinniped teeth. *Oikos* 10.1111/oik.04441 (2017).

2. Bennett, K. A. *et al.* Effects of age and body mass on development of diving capabilities of gray seal pups: costs and benefits of the postweaning fast. *Physiol. Biochem. Zool.* **83,** 911–923 (2010).

3. SCOS. *Scientific advice on matters related to the management of seal populations: 2012*. *SCOS Main Advice Report* (2012).

4. Ramasco, V., Barraquand, F., Biuw, M., McConnell, B. & Nilssen, K. T. The intensity of horizontal and vertical search in a diving forager: the harbour seal. *Mov. Ecol.* **3,** 15 (2015).
